# The role of dopamine in dynamic effort-reward integration

**DOI:** 10.1038/s41386-020-0669-0

**Published:** 2020-04-08

**Authors:** Jochen Michely, Shivakumar Viswanathan, Tobias U. Hauser, Laura Delker, Raymond J. Dolan, Christian Grefkes

**Affiliations:** 1 Wellcome Centre for Human Neuroimaging, University College London, London, WC1N 3BG UK; 2 Max Planck UCL Centre for Computational Psychiatry and Ageing Research, University College London, London, WC1B 5EH UK; 3 Medical Faculty, University of Cologne and Department of Neurology, University Hospital Cologne, 50937 Cologne, Germany; 4 Cognitive Neuroscience, Institute of Neuroscience & Medicine (INM-3), Research Centre Juelich, 52425 Juelich, Germany

**Keywords:** Reward, Motivation, Reward

## Abstract

When deciding to act, the neurotransmitter dopamine is implicated in a valuation of prospective effort and reward. However, its role in dynamic effort-reward integration during action, a process central to everyday behaviour, remains unclear. In a placebo-controlled, within-subject, study, we probed the impact of increasing brain dopamine levels (150 mg of levodopa) and blocking dopamine receptors (1.5 mg of haloperidol) in the context of a novel dynamic effort task in healthy human subjects. We show that modulating homoeostatic dopamine balance distinctly alters implicit and explicit effort allocation as a function of instantaneous reward. Pharmacologically boosting dopamine enhanced motor vigour, reflected in an implicit increase in effort allocation for high rewards. Conversely, pharmacological blockade of dopamine attenuated sensitivity to differences in reward context, reflected in reduced strategic effort discounting. These findings implicate dopamine in an integration of momentary physical experience and instantaneous reward, suggesting a key role of dopamine in acting to maximise reward on the fly.

## Introduction

Motivation encompasses the invigorating impact that incentives exert on behaviour, reflected in an enhanced willingness to engage in effortful actions to obtain rewards [1]. Yet, to determine whether an action is worth initiating and persevering with, individuals need to integrate potential benefits with the physical costs of an action. Thus, a decision to engage in effortful behaviour reflects a critical cost-benefit trade-off, a process that appears to be awry in neuropsychiatric disorders, such as depression, schizophrenia or Parkinson’s disease [2, 3].

A common approach to assess cost-benefit valuations requires subjects to choose between actions associated with varying levels of effortful demands and varying levels of reward outcomes [4, 5]. Importantly, however, in such experiments, subjects make a decision before movement initiation whether to expend effort in future action, and rewards are discounted based on anticipated effort. In contrast, deciding how much motor vigour we should exert during an action, based on actual, experienced effort costs, appears similarly relevant in everyday behaviour [6, 7].

Previous research in rodents and humans ascribes a central role to dopamine in motivational decision-making [8, 9]. Whilst dopamine blockade and depletion reduce a willingness to choose effortful options in the service of maximising reward [10–13], boosting dopamine increases a propensity to choose high effort options associated with high reward outcomes [13–15].

However, despite ample research addressing the role of dopamine in effort-based decision-making before action initiation, there is sparse human data regarding its impact on motor vigour during an action [16, 17]. Here, it remains unclear if dopamine influences a dynamic arbitration between the benefit of instantaneous reward and the cost of current, as well as future, physical demands.

Consequently, we designed a novel reward-based motor task to characterise the role of dopamine in dynamic effort-reward integration. In this physically demanding task, subjects were asked to squeeze a grip force device to maximise reward outcomes, whilst momentary reward was changing dynamically.

First, we hypothesised that changes in instantaneous reward implicitly modulate motor vigour if an effort-reward integration is dynamic. Second, we conjectured subjects, in addition to a valuation of current states, explicitly integrate deterministic information about future reward and effort levels on the fly. Ultimately, we probed the role of dopamine in dynamic effort-reward integration, using a pharmacological modulation with dopamine enhancement (150 mg of levodopa), dopamine blockade (1.5 mg of haloperidol) and placebo, in a within-subject, study design in healthy human subjects.

## Methods

### Subjects

In total, 20 healthy volunteers (mean age: 25.6; range 21–35 years, 9 females) participated in this double-blind, placebo-controlled, within-subject study design over 3 different days. All subjects underwent an electrocardiogram to exclude QT interval prolongation, and a medical interview to exclude any neurological or psychiatric disorder, other medical conditions or medication intake.

In addition, we used self-report questionnaires, administered at the beginning of the first experimental session, to screen for depression (Beck’s Depression Inventory II [18]; mean score: 1.6, range 0–7), and to assess interindividual variability in real-life motivation (Achievement Motivation Scale [19, 20]). The study was approved by the local ethics committee, with informed consent obtained from all participants.

### Pharmacological manipulation and procedures

**Fig. 1:**
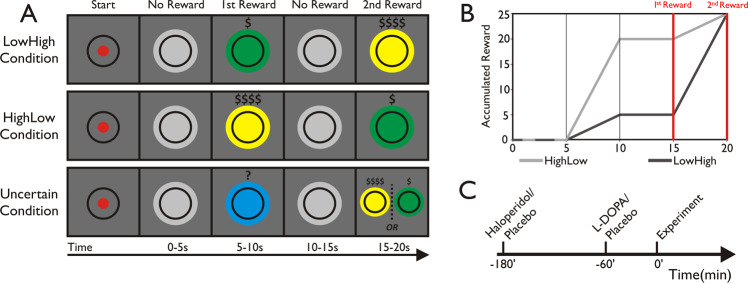
Experimental paradigm and pharmacological procedure. **a** Each trial (max. 20 s) comprised four 5-s periods. Across time periods, the balloon changed colours, indicating reward rate per second exceeding the target circle. Green indicated low reward (1 point/s), yellow indicated high reward (4 points/s), blue indicating uncertain reward (low or high, i.e., 1 point/s or 4 points/s) and grey indicating no reward. Subjects had an initial 3 s to inflate the balloon and only found out at the 5-s mark, in which condition they were in. Note that the different conditions did not vary in maximum reward, but in the reward rate over time alone. In the uncertain condition, subjects only found out about the condition when reaching the 15-s mark. **b** Subjects were awarded 25 points for completion of any trial (2nd reward endpoint). However, accumulated reward differed between conditions. Note that subjects were only awarded points for a given trial when passing the 15- s mark (1st reward endpoint) and obtained points from the early reward period plus points scored for every second exceeding the target force in the late reward period. **c** To ensure peak plasma concentrations coincided with the start of our experiment on all testing sessions, subjects received two different pills per session, 180 min and 60 min prior to the start of the experiment: levodopa session (placebo−150 mg of levodopa), haloperidol session (1.5 mg of haloperidol−placebo) and placebo session (placebo−placebo).

Subjects were tested on three sessions: once on 150 mg of levodopa, a drug acting to increase brain dopamine levels, commonly used in the treatment of Parkinson’s disease [21]; once on 1.5 mg of haloperidol, a drug predominantly blocking dopaminergic D2 receptors, commonly used in the treatment of psychosis [22]; once on placebo. Drugs used in this study have different pharmacokinetic properties, with levodopa reaching peak plasma levels after approximately 1 h, and haloperidol peaking after 3 h. To ensure that peak plasma concentrations coincided with the start of experimental testing, we used a previously described method [23, 24]. On every session, participants received two identically appearing pills, one at the beginning of the session, and one 2 h later, with the following order of administration: levodopa (placebo–levodopa), haloperidol (haloperidol–placebo) and placebo (placebo–placebo). The experiment started 1 h after the second administration, i.e., 1 h after levodopa, 3 h after haloperidol intake, respectively (Fig. 1c). Drug order (six different options) was randomly assigned on a subject-by-subject basis and was unknown to the experimenter to achieve a full double-blind design. Sessions were performed at a similar time of day, and separated by a wash-out phase of at least 1 week. Post-session evaluation, after each experimental session, demonstrated that subjects were unaware of whether they had received an active drug or placebo, confirming a successful blinding procedure (haloperidol: χ^2^_1,19_ = 0.80, *p* = 0.37; placebo: χ^2^_1,19_ = 0.20, *p* = 0.66; levodopa: χ^2^_1,19_ = 1.80, *p* = 0.18; no difference across drug conditions: all *p* > 0.34).

### Experimental paradigm

The computerised task (Fig. 1a) was implemented in Psychtoolbox-3 (www.psychtoolbox.org) for Matlab. Subjects were asked to squeeze an isometric grip force transducer (MLT004, AdInstruments Ltd, New Zealand) in order to inflate a coloured balloon shown in the centre of a computer screen. The size of the balloon changed depending on the applied force such that subjects received immediate visual feedback about their current force level. To obtain rewards, subjects had to ensure that the size of the balloon exceeded a black target circle. Every trial lasted for a maximum of 20 s, divided into four 5-s periods, between which the balloon changed colours, indicating reward rate per second exceeding the target circle.

In the first period of a given trial, subjects had an initial 3 s to inflate the balloon to ensure that it exceeded the target force. In the first (0–5 s) and third period (10–15 s), the balloon was of grey colour, indicating no reward could be earned. In the second (5–10 s) and fourth period (15–20 s), a balloon colour change to yellow indicated high reward, i.e., 4 points/s, whereas a change to green indicated low reward, i.e., 1 point/s.

The task comprised three different conditions, differing in the order of reward periods. Critically, the maximum reward attainable per trial (25 points) was held constant across conditions, but conditions varied in the accumulated reward over time, and visual cues about reward rates. On a given trial (Fig. 1a), subjects found out about the current reward condition at the 5-s mark, indicated by a corresponding colour change of the balloon for the early reward. In the ‘HighLow’ condition, early reward was high, and late reward low, whereas in the ‘LowHigh’ condition, the order was reversed, i.e., low reward early, high reward late. In an ‘uncertain’ condition, the balloon was blue in colour during the early reward, and uninformative to subjects as to the current reward rate. If the last reward indicated high reward, then, in retrospect, the first one had been low reward, and vice versa. Thus, in uncertain trials, blue colour indicated either 4 points/s or 1 point/s. Here, late reward was not predicted by early reward; thus, strategic action preparation was not possible as subjects only found out about the current condition at the 15-s mark. Note that the motivation behind the inclusion of an ‘uncertain’ condition was twofold. First, it allowed examining strategic effort discounting. Note that late reward was always of either high or low value. The deterministic nature of the ‘certain’ conditions enabled subjects to predict late reward value in advance. In contrast, early reward was uninformative in the ‘uncertain’ condition, thus precluding strategic preparation. Thus, comparing the two allowed us to examine the extent to which deterministic information influenced subjects’ strategic effort discounting. Second, in the early reward period, the uncertain condition signalled intermediate reward (as reward was, on average, the mean of high and low), whilst also signalling uncertainty, allowing a more specific assessment of cue-induced force change at the 5-s mark, over and above a dissociation between high and low reward.

Critically, subjects were informed that points were only awarded after reaching the first reward endpoint, i.e., the 15-s mark. Thus, if subjects did not pass this mark, no points were gained for a given trial. However, if subjects passed the mark, they obtained points earned from the early reward period plus points for every second exceeding the target force in the late reward period (Fig. 1b).

Overall, subjects performed 48 trials, 16 per condition. Target force levels, i.e., force required to exceed the target circle, were adjusted to 50% maximum grip force (range: 48.5–51.5%) for each subject and hand separately. Trial length and target force levels were set after extensive piloting to ensure that subjects reached the 15-s mark on most of the trials (allowing appropriate analysis of motor vigour), yet difficult enough such that a strategic player would be incentivised to discount rewards for exerting further effort after reaching the first reward (allowing appropriate analysis of strategic effort discounting). Subjects were asked to switch hands for each trial, and were allowed 20 s of rest between trials, and two 3-min breaks after completing 1/3 and 2/3 of the experiment.

### Motor vigour analysis

We hypothesised that reward rate differences across conditions would modulate motor vigour if a cost-benefit valuation was dynamic. We conjectured that subjects would apply more force to secure high as compared with low rewards. Critically, we predicted this effect despite subjects being informed that their score only depended on time spent exceeding the target circle, but not on how much they exceeded the target circle through exertion of additional force.

Motor vigour was operationalised as mean applied force, with which subjects exceeded the respective force threshold in response to the early reward, i.e., 5–15 s. As we were interested in force changes on slow multi-second timescales, grip force data were smoothed with a 300-ms sliding average to attenuate the impact of high-frequency fluctuations in muscle contraction and measurement artefacts. As we were interested in a cue-induced, event-related force change, grip force data were baseline-corrected [25, 26]. We used a 1-s baseline period preceding the reward cue, in a trade-off between avoiding inclusion of the initial force ramp-up and achieving a steady-state baseline average. Following similar standardisation procedures in grip/response force studies, e.g. [7, 17, 27], and in order to correct for differences in mean force and variance, grip force (recorded with 400 Hz) was z-scored for every subject and session, across trials, for each timepoint. This was implemented in order to bring force values onto a common scale that allowed accurate comparison between reward conditions.

Note that grip force analysis was restricted to the 5–15-s period because an analysis of the 15–20-s period would be confounded as many trials were not completed in their entirety, resulting in a relatively low number of trials. This lack of completion additionally differed between conditions due to effort discounting effects as described below.

### Effort discounting analysis

We hypothesised that effort discounting would result in subjects exerting less effort after reaching the first reward, when low compared with high reward was at stake. Consequently, strategic effort discounting was operationalised as the time subjects succeeded to stay above the target threshold in the late reward period (15–20 s), as a function of reward (high/low) and predictability (certain/uncertain). Note that 5 s denotes the maximum, i.e., an entirely completed late reward period.

### Maximum grip force and fatigue analysis

To assess maximum grip force, subjects were tasked to squeeze a grip force device with maximum intensity over four trials with either hand. Average peak force of the three best trials was computed for each hand separately and used as a calibration force to adjust target force levels to subjects’ individual capacity in the task [6, 28]. In order to eliminate any drug effects on this measure and to avoid a confound of the maximum force assessment after being aware that task difficulty would be adapted to this measure, we used the day-one baseline assessment to adjust experimental force levels on all three sessions.

To examine potential drug effects on objective fatigue, we repeated the force assessment before and after completion of the experiment on each session. To assess subjective sensation of fatigue, we asked subjects to provide a rating on the Borg scale, a common measure for assessing subjective perception of physical fatigue [29–31], during breaks and after task completion.

### Statistical analysis

Drug effects on task performance and fatigue were assessed using repeated measures (rm-)ANOVA with factor ‘drug’ (levodopa/haloperidol/placebo), and factors ‘reward’ (motor vigour: high/low/uncertain; effort discounting: high/low) or ‘time’ (objective fatigue: pre-/post; subjective fatigue: 1st/2nd/3rd). Note that the effort discounting analysis comprised an additional factor ‘certainty’ (certain/uncertain), depending on whether late reward was predictable or not. Follow-up paired *t* tests were used to further explore significant interactions between drug and reward value. Finally, we assessed the relationship between dopaminergic task effects and interindividual variability in real-life motivation, where we conjectured that subjects with lower motivation would be more prone to an interference of a homoeostatic dopamine balance (correlation analyses, one-tailed).

### Control analyses

First, we assessed drug effects on overall task performance, comparing (i) completed trials, (ii) mean time above target threshold and (iii) total score across drug conditions. Subjects completed a similar proportion of trials, maintained target force for a similar time per trial and achieved a similar score, without any significant differences across drug conditions (all *p* > 0.79; Supplementary Table S1).

Second, as dynamic effort-reward integration could only take place after reaching the first reward period at 5 s, trials terminating before that point were discarded from further analysis, though this was without significant differences across drug conditions (all *p* > 0.29; Supplementary Table S1).

Third, we assessed whether a dopaminergic manipulation impacted early effort discounting, operationalised in terms of subjects’ likelihood of reaching the first reward endpoint at 15 s. We show that participants reached the first reward endpoint more often in the HighLow condition, with no difference between drugs (all *p* > 0.55; Supplementary Table S1).

## Results

### Boosting dopamine levels enhances implicit motor vigour

**Fig. 2:**
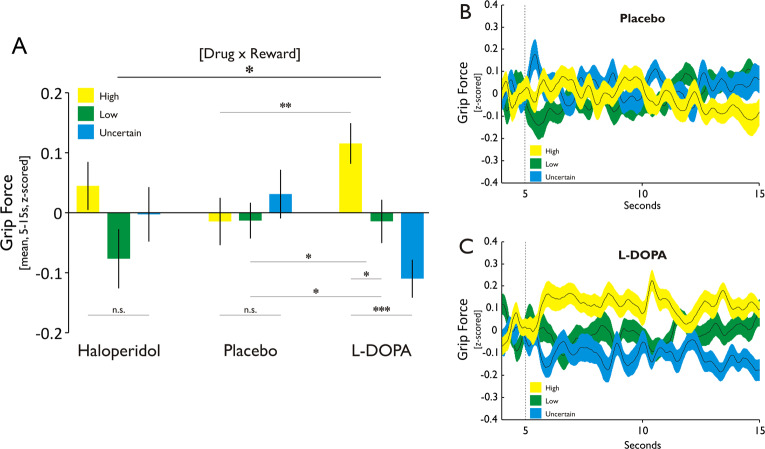
Levodopa enhances motor vigour as a function of instantaneous reward. **a** Mean applied force with which subjects exceeded target force in the early reward period (5–15 s). Under levodopa, subjects applied significantly more force in pursuit of high, compared with low and uncertain, rewards. This effect was absent under placebo and haloperidol. Moreover, the difference between high vs. low, and high vs. uncertain, was significantly larger under levodopa compared with placebo. Ultimately, applied force for high rewards was significantly enhanced under levodopa vs. placebo. ***p < 0.001, **p < 0.01, *p < 0.05, n.s. = not significant. Error bars = SEM. **b** Time course of applied force as a function of reward for placebo. **c** Time course of applied force as a function of reward for levodopa. Note that data for levodopa and placebo in (**a**) represent the mean of (**b**) and (**c**) for the 5–15-s period, respectively.

We first assessed whether dopamine modulated motor vigour, measured by mean applied force with which subjects exceeded the respective force threshold to reach the first reward endpoint (note that the control analysis above showed no drug effects on how likely subjects reached that endpoint). We found no significant effect of reward [*F*_2,38_ = 1.92, *p* = 0.16], but a significant interaction between drug and reward [*F*_4,76_ = 3.18, *p* = 0.018]. Follow-up tests revealed that subjects, in the levodopa session, applied significantly more force in the high, compared with the low and uncertain, reward condition (high vs. low: *t*_19_ = 2.09, *p* = 0.049; high vs. uncertain: *t*_19_ = 4.01, *p* < 0.001; Fig. 2a, c). These effects were absent in the placebo (high vs. low: *t*_19_ = −0.02, *p* = 0.98; high vs. uncertain: *t*_19_ = −0.61, *p* = 0.55; Fig. 2a, b), and haloperidol sessions (high vs. low: *t*_19_ = 1.57, *p* = 0.13; high vs. uncertain: *t*_19_ = 0.65, *p* = 0.52). In addition, we found grip force for high reward being enhanced under levodopa compared with placebo (*t*_19_ = 3.43, *p* = 0.003; Fig. 2a), with no such effect under haloperidol (*t*_19_ = 1.14, *p* = 0.27). To rule out the possibility that the results were affected by order of drug administration, we computed a control analysis with drug order as an additional between-subject factor. This analysis revealed no significant interaction (‘drug’ × ‘reward’ × ‘drug order’: *F*_20,56_ = 1.47, *p* = 0.13), showing that drug order did not affect the presented finding.

Subjects were informed that their overall score was independent of how much they exceeded the target circle by exerting additional force. In line with that, strategy debriefing, following the completion of the last session, revealed that no subject (0/20) reported squeezing the grip force device with more intensity to obtain high rewards, suggesting that reward-evoked force changes reflected an implicit process.

Overall, despite reaching the first reward endpoint equally often across drug conditions, subjects under levodopa implicitly applied more force in order to secure high rewards. This result is consistent with a boosting of dopamine increasing motor vigour in the context of high reward.

### Blocking dopamine receptors attenuates strategic effort discounting

**Fig. 3:**
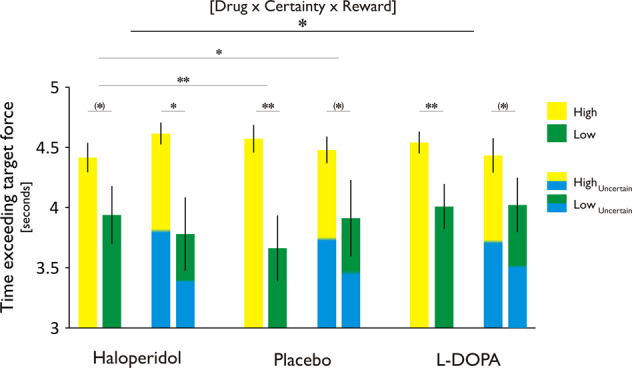
Haloperidol attenuates strategic effort discounting. Strategic effort discounting was operationalised as the time subjects succeeded to stay above the target threshold in the late reward period (15–20 s), as a function of reward (high/low) and predictability (certain/uncertain). Note that 5 s denotes the maximum, i.e., an entirely completed late reward period. Overall, subjects were more likely to sustain effort in the late reward period when a high, as compared with a low, reward was at stake. In the haloperidol session, subjects dissociated significantly less between high and low reward as compared with the placebo session. Critically, this reduced sensitivity to differences in reward context was only evident in certain conditions, where late reward value was deterministically predicted by early reward, allowing for strategic action preparation. ***p < 0.001, **p < 0.01, *p < 0.05, (*) *p* < 0.1. Error bars = SEM.

Next, we examined whether dopaminergic modulation impacted strategic effort discounting, operationalised as the time subjects succeeded to stay above the target threshold in the late reward period, as a function of reward (high/low) and predictability (certain/uncertain). Here, we found a highly significant effect of reward [*F*_1,19_ = 8.86, *p* = 0.008], indicating that subjects were more likely to sustain effort in the late reward period when a high, as compared with a low, reward was at stake (placebo: *t*_19_ = 3.68, *p* = 0.002; levodopa: *t*_19_ = 2.87, *p* = 0.010; haloperidol: *t*_19_ = 1.87, *p* = 0.076). In addition, we found a significant three-way interaction between drug, reward and certainty [*F*_2,38_ = 3.26, *p* = 0.049; Fig. 3]. This effect was driven by the fact that subjects, after dopamine blockade, dissociated significantly less between high and low reward as compared with the placebo session. Critically, this was only the case in the certain (*t*_19_ = 2.89, *p* = 0.009), but not in the uncertain condition (*t*_19_ = 1.05, *p* = 0.31). Accordingly, the dissociation between high and low reward in the certain vs. the uncertain condition was significantly different between haloperidol and placebo (*t*_19_ = 2.50, *p* = 0.022), whereas such an effect was not present under levodopa (*t*_19_ = 0.84, *p* = 0.41), forming the basis for the significant three-way interaction. Note that the drug effect was not due to a significant difference between haloperidol and placebo for high (*t*_19_ = 1.1, *p* = 0.29), or low reward (*t*_19_ = 1.4, *p* = 0.17), but rather the difference between the two, i.e., high vs. low (*t*_19_ = 2.9, *p* = 0.009). Thus, under the influence of haloperidol, subjects were less sensitive to differences in reward context. Note that the result was not affected by order of drug administration (‘drug’ × ‘certainty’ × ‘reward’ × ‘drug order’: *F*_10,28_ = 1.46, *p* = 0.21).

In the strategy debriefing following the last session, 15/20 subjects explicitly reported having tried less hard, or even stopping, when a low as compared with a high reward was at stake in the late reward period, suggestive of a more strategic, explicit effect. Owing to the deterministic nature of the ‘certain’ conditions, subjects were enabled to predict late reward value in advance. In contrast, in the ‘uncertain’ condition, late reward value was only revealed after reaching the first reward endpoint. Thus, our result suggests that haloperidol reduced a dissociation in effort allocation for distinct reward levels, and this effect was only evident when subjects were able to explicitly anticipate upcoming reward values and plan actions strategically.

Overall, these findings suggest subjects’ integrated costs and benefits of actions explicitly, as depicted by an increased effort discounting for low, compared with high, rewards. Critically, blocking dopamine receptors with haloperidol attenuated explicit effort discounting, particularly when deterministic information about expected rewards was provided in advance.

### No drug effect on subjective and objective fatigue

Next, we assessed putative drug effects on objective (pre- and post-experiment maximum grip force) and subjective (Borg scale ratings given throughout the experiment) measures of fatigue. Here, we found a significant effect of time for both objective and subjective measures, but no effect of drug, or any interaction of drug and time (Supplementary Table S2). This indicates that subjects became objectively, as well as subjectively, more fatigued over the course of the experiment, but fatigue was unaffected by pharmacological modulation.

### The relationship between dopaminergic effects and real-life motivation

Finally, we assessed whether interindividual variability in real-life motivation related to drug effects in our task. Thus, we correlated self-reports of real-life motivation with the effect of dopamine blockade on explicit effort discounting (haloperidol vs. placebo), and the effect of dopamine on implicit motor vigour (levodopa vs. placebo), respectively. We found a significant negative correlation between real-life motivation and haloperidol effects on effort discounting (ρ = −0.40, *p* = 0.039; Supplementary Fig. S1), indicating that subjects with low motivation displayed greater effects of dopamine blockade. There was no evidence for a relationship between self-reported motivation and levodopa effects on motor vigour (ρ = −0.12, *p* = 0.30; Supplementary Fig. S1).

## Discussion

Dopamine is considered to play a role in valuating prospective effort and reward when we make a decision to act, such as to expend effort in future action [8, 9]. Using a novel dynamic effort task, we show that modulating homoeostatic dopamine balance influences how humans allocate effort to maximise rewards on the fly.

Subjects dynamically adapted the intensity with which they pursue their current goal, as a function of momentary reward [6]. Critically, boosting dopamine, by means of levodopa, affected this dynamic process, by enhancing motor vigour (applied force) during pursuit of highly rewarding outcomes. This suggests that elevating dopamine function enables a dynamic motor adaptation in response to an environmental reward context. This finding extends previous studies showing how elevating dopamine enhances a propensity to choose effort options associated with high reward outcomes, as measured before actual motor initiation [13–15].

Zenon et al. [17] similarly demonstrated that boosting dopamine function via levodopa enhances motor vigour, yet only when exerted force is proportionally linked to rewards. In contrast, in our study, participants were not aware of having applied greater force in the highly rewarding condition. Thus, our findings suggest that increased dopamine levels enhance implicit reward sensitivity during action. In line with this latter finding, Rigoli et al. [27] recently showed that humans subliminally respond more vigorously, as indexed by elevated response force, to high, compared with low, reward cues. Here, using functional neuroimaging, the authors also showed that reward effects on motor vigour correlated with task-induced dopaminergic midbrain activity. Using a pharmacological modulation, we now show that dopamine plays a key role in enhancing motor vigour for appetitively motivated actions, an effect that is likely mediated via subcortical dopaminergic circuits [32, 33].

In contrast to an increased sensitivity to reward following a dopamine enhancement, pharmacological blockade of dopamine receptors diminished a dissociation between high and low rewards, as reflected in reduced effort discounting. Critically, this effect was evident only when deterministic information about upcoming rewards was available, enabling subjects to use explicit effort discounting strategies. This aligns with earlier studies in non-dynamic task settings, showing that dopamine blockade and depletion affect explicit effort discounting in rodents and humans, depicted by reduced willingness to choose effortful options in order to maximise reward [10–13].

Notably, an impact of dopamine blockade in our study related to interindividual variability in real-life motivation, with greater effects of haloperidol in subjects reporting low motivation. It is tempting to conjecture whether less-motivated individuals are more sensitive to the intervention due to lower baseline dopamine availability, rendering them more prone to interference of a homoeostatic dopamine balance. In addition, this suggests a putative overlap in the neurochemical mechanisms involved in motivational dysfunction across clinical disorders, such as depression, schizophrenia or Parkinson’s disease, conditions that are typically associated with aberrant dopamine function [2, 33].

A limitation of our study is that we only assessed healthy individuals. Precisely how a dopaminergic impact on the integration of physical experience and instantaneous reward relates to motivational dysfunction, will need to be targeted in future clinical studies. Critically, we did not directly measure brain dopamine availability, but note that multiple studies highlight a relationship between amotivation and interindividual variation in both dopamine availability and signalling [15, 34–36]. Ultimately, investigating the underlying neural processes of the dopaminergic effects we describe constitutes an avenue for future research, using, e.g., a combination of pharmacological intervention and functional neuroimaging of effort-reward processing [37, 38].

In conclusion, we show that dopamine is implicated in a dynamic integration of momentary physical experience and instantaneous reward. Our study provides evidence that dopamine plays a key role in acting to maximise reward on the fly, and hints at a link between dopamine function and level of motivation.

## Funding and disclosure

JM was supported by a fellowship from the German Research Foundation (MI 2158/1-1). JM and CG were additionally supported by the German Research Foundation (Clinical Research Group KFO219 ‘Basal-Ganglia-Cortex-Loops: Mechanisms of Pathological Interactions and Therapeutic Modulation’; GR 3285/5-1). CG receives additional funding from the University of Cologne Emerging Groups Initiative (CONNECT group) implemented into the Institutional Strategy of the University of Cologne and the German Excellence Initiative. TUH is supported by a Sir Henry Dale Fellowship (211155/Z/18/Z) from Wellcome Trust and Royal Society, a grant from the Jacobs Foundation (2017-1261-04), the Medical Research Foundation and a 2018 NARSAD Young Investigator grant (27023) from the Brain & Behavior Research Foundation. RJD holds a Wellcome Trust Senior Investigator award (098362/Z/12/Z). The Wellcome Centre for Human Neuroimaging is supported by core funding from the Wellcome Trust (203147/Z/16/Z). The Max Planck UCL Centre for Computational Psychiatry and Ageing Research is a joint initiative supported by the Max Planck Society and University College London. The authors declare no conflict of interest. Open access funding provided by Projekt DEAL.

## Supplementary information

Supplementary Material
