# Supplementary material for: The role of dopamine in dynamic effort-reward integration
Source: Neuropsychopharmacology. 2020 Apr 8;45(9):1448–53. doi: 10.1038/s41386-020-0669-0 (PMC7360543; doi:10.1038/s41386-020-0669-0)
Supplement: Supplementary file 1 — Supplementary Material [file 41386_2020_669_MOESM1_ESM.pdf]

Supplementary Material

	Placebo		Haloperidol		Levodopa	
	mean		mean	$P_{vs.\ Placebo}$	mean	$P_{vs.\ Placebo}$
Completion rate [reaching 20s]	0.589 ± 0.286		0.598 ± 0.250	0.886	0.593 ± 0.236	0.951
Time per trial [s]	17.39 ± 2.52		17.29 ± 2.09	0.865	17.37 ± 1.72	0.959
Total score [points]	900.9 ± 273.5		901.4 ± 221.8	0.993	883.3 ± 202.7	0.795
Failure rate [<5s]	0.039 ± 0.066		0.056 ± 0.049	0.293	0.046 ± 0.045	0.632
Completion rate [reaching15s]						
HighLow condition	0.854 ± 0.199		0.873 ± 0.143	0.711	0.852 ± 0.145	0.976
LowHigh condition	0.814 ± 0.206		0.840 ± 0.160	0.551	0.811 ± 0.149	0.955
Uncertain condition	0.811 ± 0.202		0.831 ± 0.161	0.680	0.806 ± 0.155	0.927

Supplementary Table S1. *Control analyses.*

Control analyses showed no difference across drug conditions for overall measures of performance, such as overall completion rate (across all conditions), mean time above target threshold per trial (across all conditions), or total score.

Moreover, there was no difference in trials terminating before reaching the first reward period at 5s. Note that subjects could only find out about the current reward condition of a given trial when reaching this mark.

Finally, dopaminergic manipulation did not affect early effort discounting, i.e., how likely subjects reached the first reward endpoint at 15s.

	'drug'		'time'		'drug' x 'time'
**Objective fatigue** *[pre-post maximum force]*	$F_{2,38}$=0.43 *P=0.65*		$F_{1,19}$=58.33 *P<0.001*		$F_{2,38}$=1.64 *P=0.21*
**Subjective fatigue** *[Borg ratings, 1st/2nd/3rd]*	$F_{2,38}$=2.43 *P=0.10*		$F_{2,38}$=155.69 *P<0.001*		$F_{4,76}$=0.60 *P=0.66*

Supplementary Table S2. *No drug effect on subjective and objective fatigue.*

There was a significant effect of time for both objective (pre-, post-experiment maximum force) and subjective (Borg ratings given after the 1st, 2nd, and 3rd part of the experiment) measure of fatigue, but no effect of drug, or any interaction of drug and time. This indicates subjects became objectively, as well as subjectively, more fatigued over the course of the experiment, but fatigue was unaffected by pharmacological modulation.

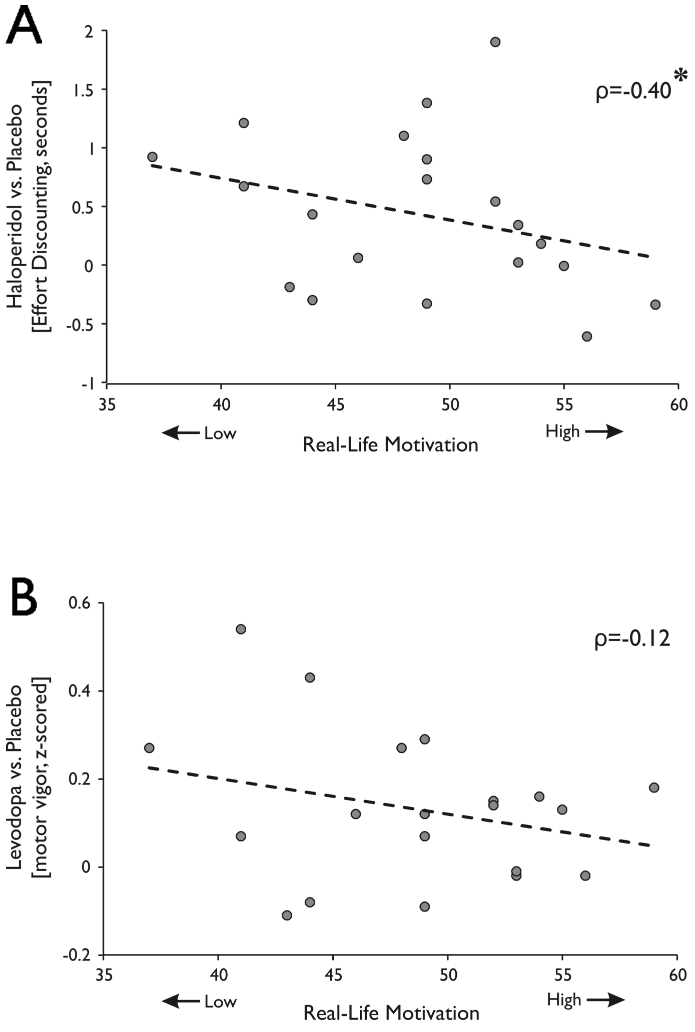

Supplementary Figure S1. *Effects of haloperidol, but not levodopa, relate to real-life motivation.*

(A) Lower self-reported motivation was associated with greater effects of haloperidol on effort discounting (group effect, cf. Fig. 3, main manuscript). * $p < 0.05$.

(B) There was no significant relationship between motivation levels and the effects of levodopa on motor vigour (group effect, cf. Fig. 2, main manuscript).
